# Motivations for weight loss in adolescents with overweight and obesity: a systematic review

**DOI:** 10.1186/s12887-018-1333-2

**Published:** 2018-11-21

**Authors:** David Franciole Oliveira Silva, Karine Cavalcanti Maurício Sena-Evangelista, Clélia Oliveira Lyra, Lucia Fátima Campos Pedrosa, Ricardo Fernando Arrais, Severina Carla Vieira Cunha Lima

**Affiliations:** 1 Postgraduate Program in Nutrition, Federal University of Rio Grande do Norte, Avenida Senador Salgado Filho, 3.000, Campus Universitario, Lagoa Nova, Natal, RN 59.058-970 Brazil; 2 Department of Nutrition, Federal University of Rio Grande do Norte, Natal, Brazil; 3 Department of Pediatrics, Pediatric Endocrinology Unit, Federal University of Rio Grande do Norte, Rua Gal. Cordeiro de Farias, s/n° - Petrópolis, Natal, RN 59012-570 Brazil

**Keywords:** Obesity, Motivation, Weight loss, Adolescent, Review

## Abstract

**Background:**

Adolescents with overweight and obesity report various motivations for weight loss other than the desire for better health. However, there is little evidence regarding the main motivations for weight loss in adolescents. The present systematic review aimed to identify the motivations for weight loss in adolescents with overweight and obesity.

**Methods:**

A systematic search for original articles published up to December 2016 was carried out in the PubMed, Scopus, LILACS, and ADOLEC databases. The terms used in the search were: motivation, motive, reason, “weight loss,” “lose weight,” and adolescent.

**Results:**

Six studies (all cross-sectional) met the selection criteria and were included in the review. The instruments used to assess the participants’ motivations for weight loss were interviews and questionnaires with open questions. Seventeen motivations for weight loss were identified, the main ones being better health, esthetic/cosmetic reasons, improvements in self-esteem, and avoidance of provocation/bullying.

**Conclusions:**

The results of the present review show the need for validated instruments to assess the motivations for weight loss in adolescents with overweight and obesity. Moreover, the high frequency of motivations for weight loss related to appearance and social acceptance evidences the need for multidisciplinary weight loss interventions that consider not only the biological factors, but also the psychological and social aspects.

## Background

The prevalence of overweight and obesity in adolescents has increased significantly in the past decades [1]. For instance, a pooled analysis of 2416 population-based studies found that the prevalence of obesity had increased from 0.7% in 1975 to 5.6% in 2016 in adolescent girls, and from 0.9% in 1975 to 7.8% in 2016 in adolescent boys [2]. Recent systematic reviews have also noted the high prevalence of overweight and obesity among adolescents in various regions of the world [3, 4].

Excess weight is a risk factor for several chronic non-communicable diseases (CNCDs), such as diabetes and cardiovascular disease, both during adolescence and adulthood [5, 6]. In addition, adolescents with overweight and obesity are at higher risk of becoming adults with obesity than normal-weight adolescents, a phenomenon known as “tracking” [7]. Data from the National Longitudinal Study of Adolescent Health showed that, while only 5% of eutrophic adolescents became adults with morbid obesity (body mass index [BMI] > 40 kg/m^2^), 40% of adolescents with obesity became adults with morbid obesity [8].

Considering the physical, psychological, and social health problems related to overweight and obesity in adolescents in the short, medium and long term, the reduction and control of body weight is an important measure for the prevention and/or treatment of CNCD, as well as to obtain better quality of life and health [5, 6]. However, adolescents often seek to reduce and control their body weight for other reasons; namely, appearance and acceptance by peers [9, 10].

Identification of the motivations for weight loss in adolescents with overweight and obesity will help health professionals to define better treatment strategies for weight control in adolescents, reinforcing the importance of weight control for health and quality of life. To the best of our knowledge, the motivation for weight loss in adolescents with overweight or obesity has not yet been recorded, nor have validated instruments for recording the former been presented. At this point, we also refer to a systematic review by Silva et al. (unpublished data).

In this context, the present review aimed to identify the instruments for evaluation and the main motivations for weight loss in adolescents with overweight and obesity.

## Methods

### Design and protocol registration

The Meta-Analyzes of Observational Studies in Epidemiology (MOOSE) [11] and the Preferred Reporting Items for Systematic Reviews and Meta-Analyzes (PRISMA) [12] were used to plan, conduct, and compose the present systematic review.

The systematic review protocol was recorded in the International Prospective Register of Ongoing Systematic Reviews (PROSPERO) (<http://www.crd.york.ac.uk/PROSPERO/>) under No. CRD42017056528.

### Inclusion and exclusion criteria

The inclusion criteria were as follows: 1) Design: observational studies (cross-sectional and cohort); 2) Language: publications in Portuguese, English and/or Spanish; 3) Publication period: no restrictions; 4) Outcome: motivation for weight loss; 5) Population: adolescents aged 10 to 20 years. This age range was defined considering that it has been used in some studies on motivation and behaviors in adolescents [13, 14] and systematic reviews [15, 16]. Additionally, according to World Health Organization (WHO): “Age is often more appropriate for assessing and comparing biological changes (e.g. puberty), which are fairly universal” [17]. Thus, the use of a more expanded age range can be justified considering that behaviors, such as motivations for weight loss, can vary with the socio-cultural environment. Review articles were excluded.

### Information sources and searches

Electronic searches were performed in the LILACS, PubMed, Scopus, and ADOLEC databases. In the LILACS and ADOLEC databases, the following combinations of terms were used: (motivation OR motivations OR “motivational factor” OR “motivational factors” OR motive OR motives OR reason OR reasons OR predictor) AND (“weight loss” OR “lose weight” OR “body-weight reduction”) AND (adolescent OR adolescents). For the PubMed and Scopus databases, the search strategy used was: (motivation OR motivations OR “motivating factor” OR “motivating factors” OR motive OR motives OR reason OR reasons OR predictor) AND (“lose weight” OR “losing weight” OR “weight loss” OR slimming) AND (adolescent OR adolescents).

To recover unpublished records, the thesis and dissertation banks of the following institutions were searched: The Coordination for the Improvement of Higher Education Personnel, the University of São Paulo, the Brazilian Institute of Information in Science and Technology. The OpenGrey database (<http://www.opengrey.eu/>), which specializes in grey literature, was also searched, and a manual search was performed.

### Study selection and data extraction

The first author (D.S.) triaged the retrieved records, while another author (S.L.) reviewed the selected studies. After identifying the studies that would be included in the review, the researchers collected the following data: journal, language, year of publication, authorship, country of origin, number and age of participants, method of nutritional status classification, instrument for assessing motivations and main motivation for weight loss. The motivations for weight loss were compared across studies, with consideration of the similarity among the motivations. That is, studies that described similar motivations related to appearance were considered as reporting the same motivation.

### Quality appraisal

Two researchers assessed the methodological quality of the studies included in the review using the Agency for Healthcare Research and Quality (AHRQ) Methodology Checklist for Cross-Sectional/Prevalence Studies (http://www.ncbi.nlm.nih.gov/books/NBK35156/) [18]. The AHRQ checklist consists of 11 items that can be answered with either “yes”, “no”, or “unclear”. We classified studies with 8–11 “yes” responses as “high quality”; those with 4–7 were “moderate quality”, and those with 0–3 were “low quality”.

## Results

### Search outcomes

**Fig. 1:**
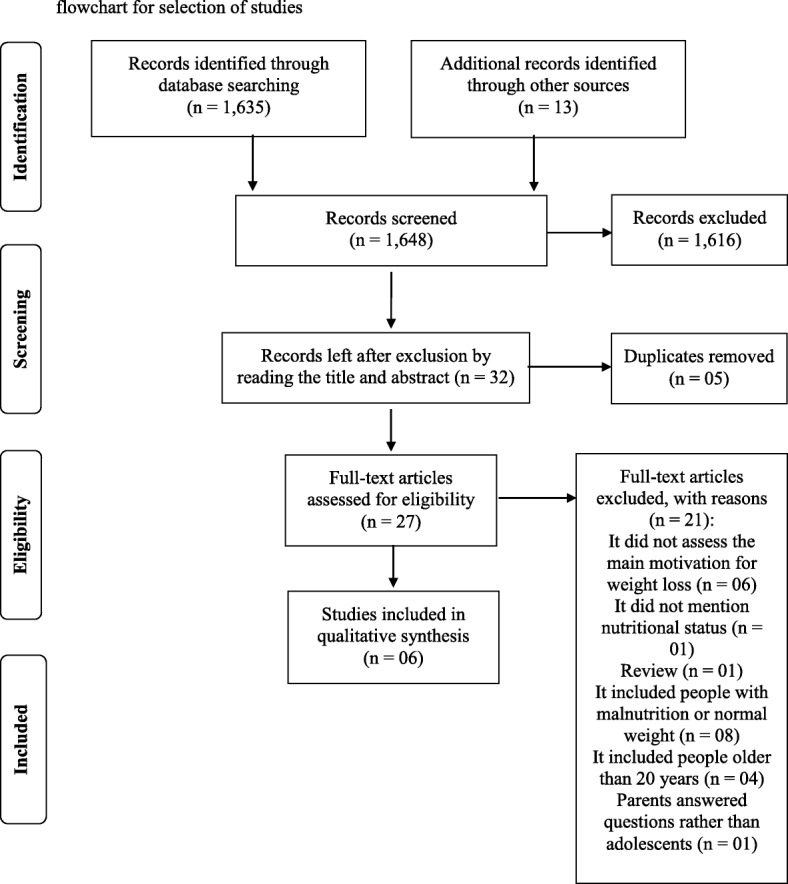
Preferred Reporting Items for Systematic Reviews and Meta-Analyzes (PRISMA) flowchart for selection of studies

The search in the PubMed, Scopus, LILACS, and ADOLEC databases retrieved 1635 records. In the manual search, 13 articles were retrieved for a total of 1648 records. After reading the titles and the abstracts, we excluded 1616 abstracts. Of the 32 abstracts selected, five were duplicates. The remaining 27 were assessed for eligibility. After the inclusion and exclusion criteria were assessed, six articles were included in the present review; all were cross-sectional studies [19–24]. Figure 1 presents the flowchart for the selection of the studies.

### Quality appraisal

The methodological quality and characteristics of the studies are presented in Table 1. Five studies were classified as having moderate quality because they received a “yes” response for 4–7 items of the AHRQ checklist [19–21, 23, 24]. One study received a “yes” response for eight items and was classified as high quality [22].

**Table 1:** Characteristics of the studies included in the review

Author (year)	Country/ Design	N	Age group, years	Nutritional status (diagnostic criteria)	Quality score
Jensen et al. (2014) [19]	United States/ Cross-sectional	40 (32 girls, 8 boys)	15–20	Overweight (≥ 85th BMI percentile in individuals aged 14–17 years or BMI ≥ 25 in individuals aged 18–20 years)	5 (Moderate)
Taylor et al. (2013) [20]	United States/ Cross-sectional	20 girls	12–19	Severely obese (≥ 150% of estimated ideal body weight)	7 (Moderate)
In-iv et al. (2010) [21]	Thailand/ Cross-sectional	167 (121 girls, 46 boys)	Mean: 14.5 ± 1.6	Mildly obese (BMI ≥ 25 kg/m^2^ + %WFH from 120 to 140%) Moderately to severely obese (BMI ≥ 25 kg/m^2^ + %WFH > 140%)	6 (Moderate)
Morinder et al. (2011) [22]	Sweden/ Cross-sectional	18 (12 girls, 6 boys)	14–16	Obese (international age- and gender-specific BMI cut-off points established by the IOTF)	8 (High)
Reece et al. (2015) [23]	United Kingdom/ Cross-sectional	12 (8 girls, 4 boys)	11–16	Overweight and obese (BMI > 91st percentile)	5 (Moderate)
Lofrano-Prado et al. (2013) [24]	Brazil/ Cross-sectional	128 (76 girls, 52 boys)	12–18	Obese (BMI ≥ 95th percentile)	4 (Moderate)

Abbreviations: *BMI* Body mass index, *N* Number of study participants, *IOTF* International obesity task force, *%WFH* Weight-for-height percentage

### Study characteristics

Two studies were conducted in the United States of America [19, 20], one in Thailand [21], one in Sweden [22], one in the United Kingdom [23], and one in Brazil [24]. The range of sample sizes varied between 12 [23] and 167 [21] adolescents; whereas the age range went from 11 [23] to 20 [19] years.

The most commonly used diagnostic criterion for overweight and obesity among the studies was that of the Center for Disease Control (percentile of BMI by age), which was used in two studies [19, 24]. Other studies established different criteria and, in particular, did not define (extreme) obesity uniformly. Morinder et al. [22], for example, used the International Criteria of the Obesity Task Force, whereas In-Iw et al. [21] applied the following criteria: slightly obese - BMI ≥ 25 kg/m^2^ combined with a weight between 120 and 140%, and moderately-severely obese - BMI ≥ 25 kg/m^2^ combined with weight for body size percentage > 140%. Reece et al. [23] defined overweight as a BMI percentile > 91, and the study by Taylor et al. [20] classified severely obese as a weight for height percentage ≥ 150% [15]. One study included only adolescents with overweight [19], four included only adolescents with obesity [20–22, 24], and one included both [23].

### Motivations for weight loss in adolescents with overweight and obesity

**Fig. 2:**
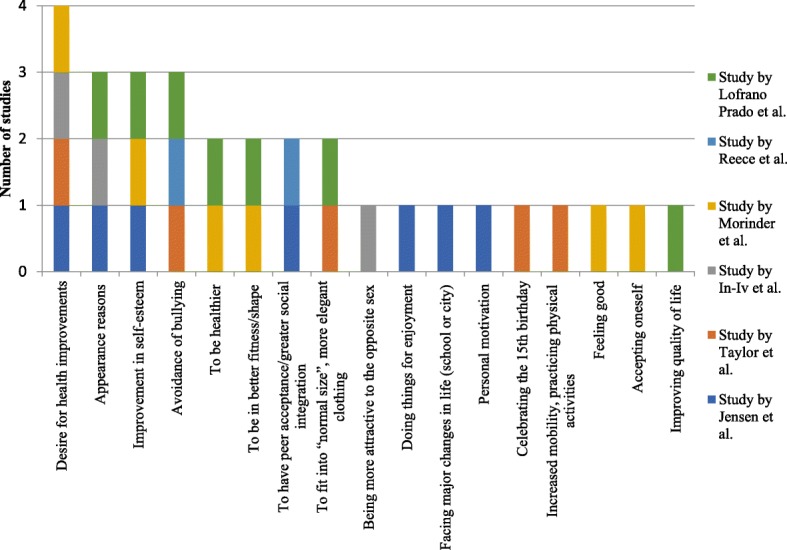
Motivations for weight loss among studies

The instruments for assessing and the motivations for weight loss among adolescents with overweight and obesity are presented in Table 2 and Fig. 2. An interview with open questions was the most frequent instrument, used in four studies [20, 22–24]. Two studies used questionnaires with open questions [19, 21]. An example of an open question used in the studies would be “What are your personal motivations for losing weight?”, which was used in the study by Lofrano-Prado et al. [24].

**Table 2:** Motivations for weight loss in adolescents with overweight and obesity

Author	Instrument for assessing the motivation for weight loss	Motivations for weight loss
Jensen et al. [19]	Questionnaire with open questions	Appearance, desire for better health, doing things to enjoy, major life transition, peer acceptance, self-motivation and self-worth
Taylor et al. [20]	Interview with open questions	Celebration symbolizing a girl’s transition to womanhood at her 15th birthday, improved health, avoidance of teasing and bullying, inability to fit into “normal” or stylish clothing, and limitations on movement, physical activity, and exercise
In-iv et al. [21]	Questionnaire with open questions	Cosmetic purposes, medical reasons, and attractiveness to the opposite sex
Morinder et al. [22]	Interview with open questions	Feeling good and accepting oneself, being healthy and in good physical shape, having more self-esteem, and not worrying about hospital visits and future diseases
Reece et al. [23]	Interview with open questions	Avoidance of bullying and a desire to integrate socially with peers
Lofrano-Prado et al. [24]	Interview with open questions	To become healthy, fit in clothes, personal appearance, bullying, self-esteem, physical fitness, and quality of life

In the six studies included in the present review, 17 different motivations for weight loss in adolescents with overweight and obesity were identified [19–24]. The desire for health improvements was the main one, observed in four studies [19–22], of which three included exclusively adolescents with obesity [20–22]. In three studies, the participants stated appearance reasons [19, 21, 24]. Improvement in self-esteem was given as a motivation in three studies [19, 22, 24], while avoidance of bullying was also mentioned in three other ones studies [20, 23, 24]. The motivations to be healthier [22, 24], to be in better fitness/shape [22, 24], to have peer acceptance and/or greater social integration [19, 23], and to fit into “normal size”, more elegant clothing [20, 24] were identified in two studies each. The following motivations were mentioned in one study each: being more attractive to the opposite sex [21], doing things for enjoyment [19], facing major changes in life (school or city) [19], personal motivation [19], celebrating the 15th birthday [20], increased mobility, practicing physical activities [20], feeling good [22], accepting oneself [22], and improving quality of life [24].

## Discussion

Adolescents are particularly vulnerable to the influence of the media and their peers [25]. In adolescents with overweight and obesity, this influence may result in motivations for weight loss related to appearance and peer acceptance goals rather than improving health and quality of life [26, 27].

Most studies in the present review presented a moderate risk of bias/methodological quality, according to the AHRQ criteria [18]. Among the observed biases was the use of non-specific and non-validated instruments to assess the motivations for weight loss in adolescents with overweight and obesity.

Several sets of diagnostic criteria for overweight were used in the studies; therefore, the nutritional status of the adolescents could not be compared between the studies. In addition, this highlights the need to adopt an international criterion to estimate, monitor and compare the prevalence of overweight in different populations and therefore, to support public health interventions.

The studies included in the present review used questionnaires with open questions [19, 21] and interviews [20, 22–24] to identify the participants’ motivations for weight loss. It should be noted that such instruments do not allow standardization of the responses and that none of the questionnaires or interviews had been validated at the time the studies were carried out. For this reason, the reproducibility and generalization of the results of these studies were compromised. It follows that there is a need for a questionnaire with adequate content and construct validity as well as internal consistency and reliability for the assessment of motivations for weight loss in adolescents with overweight and obesity. Indeed, there are six questionnaires for assessing motivations for weight loss in individuals with overweight, but none are directed exclusively towards adolescents [28–33].

In three studies from the current review, the main motivation for weight loss among adolescents with obesity was the desire for better health [20–22], perhaps because the participants knew that some of the consequences of obesity, such as dyslipidemia, hyperglycemia, and insulin resistance, require greater attention and health care than adolescent obesity itself. In the study by In-iw et al. [21], the motivation to have better health was higher in the “moderate-to-severe obesity” group of adolescents (BMI ≥ 25 kg/m^2^ combined with weight-for-height percentage of 120–140%) (52%) than in the “mild obesity” group (BMI ≥ 25 kg/m^2^ combined with weight-for-height percentage > 150%) (35%; *p* = 0.03).

In half of the studies, appearance reasons were the motivations for weight loss [19, 21, 24], especially in adolescent girls. For example, in the study by Jensen et al. [19], 19 of the 32 adolescent girls identified appearance reasons as their primary motivation for weight loss. Lofrano-Prado et al. [24] reported that 30% of adolescent girls reported appearance reasons as their main motivation for weight loss, while the rate was approximately 15% in adolescent boys, indicating that adolescent girls are under greater pressure and influence from the media and their peers to have a lean and well-defined body according to beauty standards that are disseminated as ideal [34, 35].

Self-esteem in adolescents with overweight can be compromised by social pressure. They may compare their body size with other people’s and long for the same body structure as “normal-weight” models, artists, or more popular peers [36–38]. It was for this reason that the desire for improvement in self-esteem was referenced as a motivation for weight loss in three of the studies [19, 22, 24], demonstrating the need for multidisciplinary treatments that emphasize the reality of body diversity. Adolescents are in the process of developing and discovering their own bodies. Therefore, it is important that they understand that people with the same body weight may have physical variations due to bone structure. Such knowledge would promote better body acceptance and self-esteem, and the desire to lose weight would be motivated by health concerns and not necessarily by the wish for someone else’s body type.

Teasing and bullying observed in adolescents with overweight and obesity in, especially school-aged adolescents, still occurs often, triggering low self-esteem, shame, and social withdrawal [39, 40]. Indeed, avoidance of bullying was identified as a motivation for weight loss in three studies [20, 23, 24], indicating the need for measures that combat discriminatory practices in school environment. Brown et al. [41] showed that children and adolescents who reported motivation for weight loss associated with bullying presented unhealthy eating practices such as skipping meals.

Buying clothes for people with overweight is often no easy task, since the quantity and variety of clothes available are lower than for normal-weight people, even though significant achievements have been made regarding clothing for people with overweight, with the greater prominence received by fashion plus sizes [42]. In this regard, the desire to fit “normal size” clothing, which were classified as more “elegant” by the adolescents, was identified in two studies [20, 24], one of which included only adolescent girls [20].

The study by Taylor et al. [20], conducted in United States of America, which included only adolescent girls, identified the 15th birthday party as a motivation for weight loss. This motivation was not identified in the other studies included in the review [19, 21–24], which were carried out in several regions of the world. Taylor et al. [20] suggested that the period beginning 3 years before the event, that is, after the age of 12 years, may represent a turning point for adolescent girls with overweight that initiates healthy weight-control and weight-loss behaviors that may favor lasting control of body weight. This understanding cannot be generalized, considering that this motivation was identified in only one study. Nevertheless, the beginning of puberty might certainly be regarded as a critical phase in which adolescents girls with overweight and obesity start to work intensively on controlling their body weight.

From an epidemiological point of view, the motivations for weight loss in adolescents with overweight and obesity identified in the present systematic review may inform better targeting of systematized, early, multi-professional intervention that greatly impacts weight loss and control. Such interventions may prevent and/or treat comorbidities associated with obesity over time. Adolescence is a particularly important time of life, and among the risk factors for the development/maintenance of excess weight, eating and lifestyle habits are among the most modifiable. This highlights the need for effective public policies for preventing and controlling obesity that consider the individual and socio-environmental risk factors within in an integrated, inter-sectoral context [43, 44].

There are two limitations to this study. First, is possible that not all articles on the motivation for weight loss in adolescents with overweight and obesity were included. That said, searches were carried out in the main health databases, in the gray literature, and manually. Second, the instruments for evaluating the motivation for weight loss in the studies included in the review were interviews and questionnaires with open questions, without validation, compromising the comparison between the studies. The strengths of the study were: (1) its novelty – no previous systematic reviews have been carried out on the same theme; (2) the use of combinations of specific terms in each database; and (3) the assessment of methodological quality using specific criteria.

## Conclusions

In the present review, 17 motivations for weight loss were identified in adolescents with overweight and obesity using interviews and questionnaires with open questions. A validated questionnaire to assess this variable in adolescents is necessary. The main motivations for weight loss were better health, appearance reasons, improvement in self-esteem, and avoidance of bullying. The methodological quality of most studies was moderate, according to the AHRQ checklist.

Further studies on the motivations for weight loss in adolescents with overweight and obesity are recommended. In particular, associations should be made with other variables such as socioeconomic status, schooling, urban vs. rural housing, and unhealthy behaviors such as smoking and alcoholism. In addition, further experimental studies that assess the impact of multiprofessional treatment on reorientation of motivations for weight loss in adolescents with overweight and obesity are recommended.

## Abbreviations

AHRQ: Agency for Healthcare Research and Quality
BMI: Body mass index
CNCD: Chronic non-communicable disease
CVD: Cardiovascular disease
IBICT: Brazilian Institute of Information in Science and Technology (*Instituto Brasileiro de Informação em Ciência e Tecnologia*)
MOOSE: Meta-Analyzes of Observational Studies in Epidemiology
PRISMA: Preferred Reporting Items for Systematic Reviews and Meta-Analyzes
PROSPERO: International Prospective Register of Ongoing Systematic Reviews

## References

[1] Ng M, Fleming T, Robinson M, Thomson B, Graetz N, Margono C (2014). Global, regional, and national prevalence of overweight and obesity in children and adults during 1980-2013: a systematic analysis for the global burden of disease study 2013. Lancet.

[2] Abarca-Gómez L, Abdeen ZA, Hamid ZA, Abu-Rmeileh NM, Acosta-Cazares B, Acuin C (2017). Worldwide trends in body-mass index, underweight, overweight, and obesity from 1975 to 2016: a pooled analysis of 2416 population-based measurement studies in 128.9 million children, adolescents, and adults. Lancet.

[3] Rivera JA, de Cossío TG, Pedraza LS, Aburto TC, Sánchez TG, Martorell R (2014). Childhood and adolescent overweight and obesity in Latin America: a systematic review. Lancet Diabetes Endocrinol.

[4] Niehues JR, Gonzales AI, Lemos RR, Bezerra PP, Haas P (2014). Prevalence of overweight and obesity in children and adolescents from the age range of 2 to 19 years old in Brazil. Int J Pediatr.

[5] Cobayashi F, Oliveira FL, Escrivão MA, Daniela S, Taddei JA (2010). Obesity and cardiovascular risk factors in adolescents attending public schools. Arq Bras Cardiol.

[6] Park MH, Falconer C, Viner RM, Kinra S (2012). The impact of childhood obesity on morbidity and mortality in adulthood: a systematic review. Obes Rev.

[7] Deshmukh-Taskar P, Nicklas TA, Morales M, Yang SJ, Zakeri I, Berenson GS (2006). Tracking of overweight status from childhood to young adulthood: the Bogalusa heart study. Eur J Clin Nutr.

[8] Suchindran C, North KE, Popkin BM, Gordon-Larsen P (2010). Association of adolescent obesity with risk of severe obesity in adulthood. JAMA.

[9] Cheskin LJ, Donze LF (2001). Appearance vs health as motivators for weight loss. JAMA.

[10] Vartanian LR, Wharton CM, Green EB (2012). Appearance vs. health motives for exercise and for weight loss. Psychol Sport Exerc.

[11] Stroup DF, Berlin JA, Morton SC, Olkin I, Williamson GD, Rennie D (2000). Meta-analysis of observational studies in epidemiology: a proposal for reporting. Meta-analysis of observational studies in epidemiology (MOOSE) group. JAMA.

[12] Moher D, Liberati A, Tetzlaff J, Altman DG, PRISMA Group (2009). Preferred reporting items for systematic reviews and meta-analyses: the PRISMA statement. PLoS Med.

[13] McDade TW (2001). Lifestyle incongruity, social integration, and immune function in Samoan adolescents. Soc Sci Med.

[14] Tempark T, Chatproedprai S, Wananukul S (2012). Attitudes, knowledge, and behaviors of secondary school adolescents regarding protection from sun exposure: a survey in Bangkok, Thailand. Photodermatol Photoimmunol Photomed.

[15] Magliano ES, Guedes LG, Coutinho ESF, Bloch KV (2013). Prevalence of arterial hypertension among Brazilian adolescents: systematic review and meta-analysis. BMC Public Health.

[16] Noshchenko A, Hoffecker L, Lindley EM, Burger EL, Cain CM, Patel VV (2015). Predictors of spine deformity progression in adolescent idiopathic scoliosis: a systematic review with meta-analysis. World J Orthop.

[17] WHO – World Health Organization. Age: not the whole story. http://www.who.int/maternal_child_adolescent/topics/adolescence/development/en. Accessed 16 Sept 2018.

[18] Agency for Healthcare Research and Quality. Appendix D. Quality assessment forms. Cross-sectional/prevalence study quality. http://www.ncbi.nlm.nih.gov/books/NBK35156//. Accessed 6 July 2017.

[19] Jensen CD, Duraccio KM, Hunsaker SL, Rancourt D, Kuhl ES, Jelalian E (2014). A qualitative study of successful adolescent and young adult weight losers: implications for weight control intervention. Child Obes.

[20] Taylor SA, Garland BH, Sanchez-Fournier BE, Allen KF, Doak JS, Wiemann CM (2013). A qualitative study of the day-to-day lives of obese Mexican-American adolescent females. Pediatrics.

[21] In-iw S, Manaboriboon B, Chomchai C (2010). A comparison of body-image perception, health outlook and eating behavior in mildly obese versus moderately-to-severely obese adolescents. J Med Assoc Thail.

[22] Morinder G, Biguet G, Mattsson E, Marcus C, Larsson UE (2011). Adolescents’ perceptions of obesity treatment–an interview study. Disabil Rehabil.

[23] Reece LJ, Bissel P, Copeland RJ (2015). ‘I just don't want to get bullied anymore, then I can lead a normal life’; insights into life as an obese adolescent and their views on obesity treatment. Health Expect.

[24] Lofrano-Prado MC, Hill JO, Silva HJG, de Freitas CRM, de Freitas CMSM, de Lima Ferreira MDN (2013). Reasons and barriers to lose weight: obese adolescents’ point of view. Br J Med Res.

[25] Dohnt HK, Tiggemann M (2006). Body image concerns in young girls: the role of peers and media prior to adolescence. J Youth Adolesc.

[26] Thompson JK, Shroff H, Herbozo S, Cafri G, Rodriguez J, Rodriguez M (2007). Relations among multiple peer influences, body dissatisfaction, eating disturbance, and self-esteem: a comparison of average weight, at risk of overweight, and overweight adolescent girls. J Pediatr Psychol.

[27] McCabe MP, Ricciardelli LA, Holt K (2010). Are there different sociocultural influences on body image and body change strategies for overweight adolescent boys and girls?. Eat Behav.

[28] Ames GE, Perri MG, Fox LD, Fallon EA, De Braganza N, Murawski ME (2005). Changing weight-loss expectations: a randomized pilot study. Eat Behav.

[29] Braden AL, Crow S, Boutelle K (2015). Child self-reported motivations for weight loss: impact of personal vs. social/familial motives on family-based behavioral weight loss treatment outcomes. Eat Weight Disord.

[30] Meyer AH, Weissen-Schelling S, Munsch S, Margraf J (2010). Initial development and reliability of a motivation for weight loss scale. Obes Facts.

[31] Murphy K, Brennan L, Walkley J, Reece J, Little E (2011). Primary goals for weight loss questionnaire (PGWLQ): development and psychometric evaluation in overweight and obese adults. Behav Change.

[32] Stotland S, Larocque M, Sadikaj G (2012). Positive and negative dimensions of weight control motivation. Eat Behav.

[33] Striegel-Moore RH, Wilfley DE, Caldwell MB, Needham ML, Brownell KD (1996). Weight-related attitudes and behaviors of women who diet to lose weight: a comparison of black dieters and white dieters. Obesity.

[34] Spurr S, Berry L, Walker K (2013). Exploring adolescent views of body image: the influence of media. Issues Compr Pediatr Nurs.

[35] Webb HJ, Zimmer-Gembeck MJ (2014). The role of friends and peers in adolescent body dissatisfaction: a review and critique of 15 years of research. J Res Adolesc.

[36] Helfert S, Warschburger P (2013). The face of appearance-related social pressure: gender, age and body mass variations in peer and parental pressure during adolescence. Child Adolesc Psychiatry Ment Health.

[37] Iannaccone M, D'Olimpio F, Cella S, Cotrufo P (2016). Self-esteem, body shame and eating disorder risk in obese and normal weight adolescents: a mediation model. Eat Behav.

[38] Carey RN, Donaghue N, Broderick P (2014). Body image concern among Australian adolescent girls: the role of body comparisons with models and peers. Body Image.

[39] Bacchini D, Licenziati MR, Garrasi A, Corciulo N, Driul D, Tanas R (2015). Bullying and victimization in overweight and obese outpatient children and adolescents: an Italian multicentric study. PLoS One.

[40] Van Geel M, Vedder P, Tanilon J (2014). Are overweight and obese youths more often bullied by their peers? A meta-analysis on the relation between weight status and bullying. Int J Obes.

[41] Brown CL, Skelton JA, Perrin EM, Skinner AC (2016). Behaviors and motivations for weight loss in children and adolescents. Obesity.

[42] Marcuzzo M, Pich S, Dittrich MGA (2012). Construction of body image among obese subjects and its relationship with the contemporary imperatives for body beautification. Interface.

[43] Castro IRR (2017). Obesity prevention and control: the urgent need for effective public policies. Cad Saúde Pública.

[44] Dias PC, Henriques P, Anjos LA, Burlandy L (2017). Obesity and public policies: the Brazilian government’s definitions and strategies. Cad Saúde Pública.

